# Early versus delayed mobilization for in-hospital mortality and health-related quality of life among critically ill patients: a systematic review and meta-analysis

**DOI:** 10.1186/s40560-019-0413-1

**Published:** 2019-12-09

**Authors:** Yohei Okada, Takeshi Unoki, Yujiro Matsuishi, Yuko Egawa, Kei Hayashida, Shigeaki Inoue

**Affiliations:** 1 Department of Primary Care and Emergency Medicine, Graduate School of Medicine, Kyoto University, Syogoin Kawaramachi 54, Sakyo, Kyoto, 606-8507 Japan; 2 Preventive Services, School of Public Health in the Graduate School of Medicine, Kyoto University, Kyoto, Japan; 3 School of Nursing, Sapporo City University, Sapporo, Japan; 4 Emergency and Intensive Care Laboratory, Pediatric Intensive Care Unit, University of Tsukuba Hospital, University of Tsukuba, Ibaraki, Japan; 5 Advanced Emergency and Critical Care Center, Saitama Red Cross Hospital, Saitama, Japan; 6 The Feinstein Institutes for Medical Research, Department of Emergency Med-Cardiopulmonary, North Shore University Hospital, Northwell Health System, Manhasset, USA; 7 Department of Disaster and Emergency Medicine, Graduate School of Medicine, Kobe University, Kobe, Japan

**Keywords:** Early mobilization, Rehabilitation, Physiotherapy, Occupational therapy, Critical care

## Abstract

**Background:**

This systematic review and meta-analysis of randomized clinical trials aimed to investigate the efficacy of early mobilization among critically ill adult patients.

**Methods:**

We searched CENTRAL, MEDLINE, and Igaku-Chuo-Zasshi (a Japanese bibliographic database) databases until April 2019 and included randomized control trials to compare early mobilization started within 1 week of intensive care unit (ICU) admission and earlier-than-usual care with the usual care or mobilization initiated later than the intervention. Two authors independently extracted the data of the included studies and assessed their quality. The primary outcomes were in-hospital mortality, length of ICU/hospital stay, and health-related quality of life (QOL).

**Results:**

Among 1085 titles/abstracts screened, 11 studies (including 1322 patients) were included in the meta-analysis, which was conducted using the random-effects model. The pooled relative risk for in-hospital mortality comparing early mobilization to usual care (control) was 1.12 (95% CI [confidence interval]: 0.80 to 1.58, *I*^2^ = 0%). The pooled mean differences for duration of ICU and hospital stay were -1.54 (95% CI: -3.33 to 0.25, *I*^2^ = 90%) and -2.86 (95% CI: -5.51 to -0.21, *I*^2^ = 85%), respectively. The pooled mean differences at 6 months post-discharge, as measured by the Short Form 36-Item Health Survey and Euro-QOL EQ-5D, were 4.65 (95% CI: -16.13 to 25.43, *I*^2^ = 86%) for physical functioning and 0.29 (95% CI: -11.19 to 11.78, *I*^2^ = 66%) for the visual analog scale.

**Conclusions:**

Our study indicated no apparent differences between early mobilization and usual care in terms of in-hospital mortality and health-related QOL. Detailed larger studies are warranted to evaluate the impact of early mobilization on in-hospital mortality and health-related QOL in critically ill patients.

**Trial registration:**

PROSPERO (identifier CRD42019139265)

## Background

Early mobilization and physiotherapy have been used to prevent post-intensive care syndrome and ICU-acquired weakness (ICU-AW) in critically ill adult patients in the intensive care unit (ICU) [1, 2]. Two systematic reviews with meta-analysis have demonstrated that these interventions may help improve short-term physical function and decrease the duration of mechanical ventilation or ICU stay; this has also been advocated in clinical practice guidelines [1–4]. However, previous studies do not indicate whether these interventions improved more relevant outcomes such as mortality and health-related quality of life (QOL) [1–5]. Therefore, it is necessary to focus on these outcomes when examining the effects of early mobilization in ICU patients. This systematic review and meta-analysis aimed to investigate the efficacy on the mortality and health-related QOL of early mobilization among critically ill adult patients.

## Methods

We followed the Preferred Reporting Items for Systematic Reviews and Meta-Analyses (PRISMA) protocol for randomized clinical trials (RCTs) [6]. This review protocol was submitted to the International Prospective Register of Systematic Reviews (PROSPERO) on June 18, 2019, before the initiation of data extraction and was approved for registration on August 15, 2019 (PROSPERO identifier: CRD 42019139265). The protocol is described in the Additional file 1.

### Data sources and searches

We searched for eligible trials in the Cochrane Central Register of Controlled Trials (CENTRAL) via the Cochrane Library (on April 24, 2019), in MEDLINE via PubMed (on April 28, 2019), and in Igaku-Chuo-Zasshi (Ichu-shi) (on April 26, 2019). Ichu-shi is a Japanese bibliographic database managed by the Japan Medical Abstracts Society. Our search strategies are described in the Additional file 1. We also performed a manual search to retrieve all potentially relevant articles on June 21, 2019. Searches were restricted to articles written and published in English or Japanese.

### Eligibility criteria

We set the following eligibility criteria: study design was RCT, and the target populations were critically ill adult patients (aged≥18 years) admitted to the ICU. The intervention was early mobilization defined as (1) physical and/or occupational therapy, (2) started within 1 week of ICU admission, and (3) initiated earlier than usual care or control, based on the previous literature [4]. The control was defined as usual care or mobilization started later than the intervention.

### Selection of studies

Two authors (YO and YM) independently screened the titles and abstracts of the studies identified by the search strategy for inclusion eligibility and performed a hand search to identify relevant studies. Differences were resolved by discussion with other authors (TU and YE). We retrieved the full text of all possible eligible studies for further evaluation for inclusion. Two authors (YO and YE) independently assessed the full-text studies identified in the primary screening, and the eligible studies were finally identified. We provide the number of records assessed and excluded at each stage and the reasons for excluding full-text studies in the PRISMA flowchart [6].

### Data extraction and management

The authors independently extracted data from identified eligible trials using a specifically pre-designed sheet. We compared the extracted data for differences, and disagreements were resolved by discussion among authors.

### Outcomes

The primary outcomes were in-hospital mortality, length of ICU/hospital stay, and health-related QOL. Health-related QOL is defined as QOL assessed by the Short Form Health Survey 36-Item (SF-36) or EuroQol 5 dimension (EQ-5D), the two most widely accepted standardized instruments for assessing health-related QOL [7, 8]. The secondary outcomes were physical function, cognitive function, mental disorder such as depression or anxiety, and all adverse events. Physical function was assessed by grip strength, Medical Research Council (MRC) Scale for Muscle Strength score, or the Physical Function in ICU Test (PFIT) [9, 10]. Cognitive function after discharge was measured by the Mini-Mental State Examination (MMSE) [11]. For dichotomous outcomes, the total number of events and number of events within each randomization group were pooled to calculate risk ratios (RRs) with 95% confidence intervals (CIs) using random-effects models. For continuous outcomes, the median and standard deviation in each group were pooled to estimate the mean difference (MD) with 95% CI. Statistical significance was defined as the absence of overlap of a 95% CI with the null effect value (risk ratio [RR]=1).

### Assessment of risk of bias

We used the Cochrane Collaboration risk of bias tool to assess the methodological quality and the extent of potential bias of the included studies [12]. The authors independently assessed the risk of bias as “low,” “high,” or “unclear” for all domains and presented our assessment in a risk of bias table within the review. Any disagreements were resolved by discussion among authors.

### Patient and public involvement

As the study was a systematic review of the publication data, patients and/or the public were not involved.

### Statistical analysis

We used Cochrane Statistical Package Review Manager 5.3 (Cochrane Collaboration, London, UK) for data synthesis and analysis. Because we anticipated heterogeneity among studies, we conducted the meta-analysis using the random-effects model. Heterogeneity was assessed using Chi^2^ and *I*^2^ statistics. Because more than ten studies were included, we examined a funnel plot to assess the potential for publication bias for our primary outcomes [12].

## Results

### Search results

**Fig. 1:**
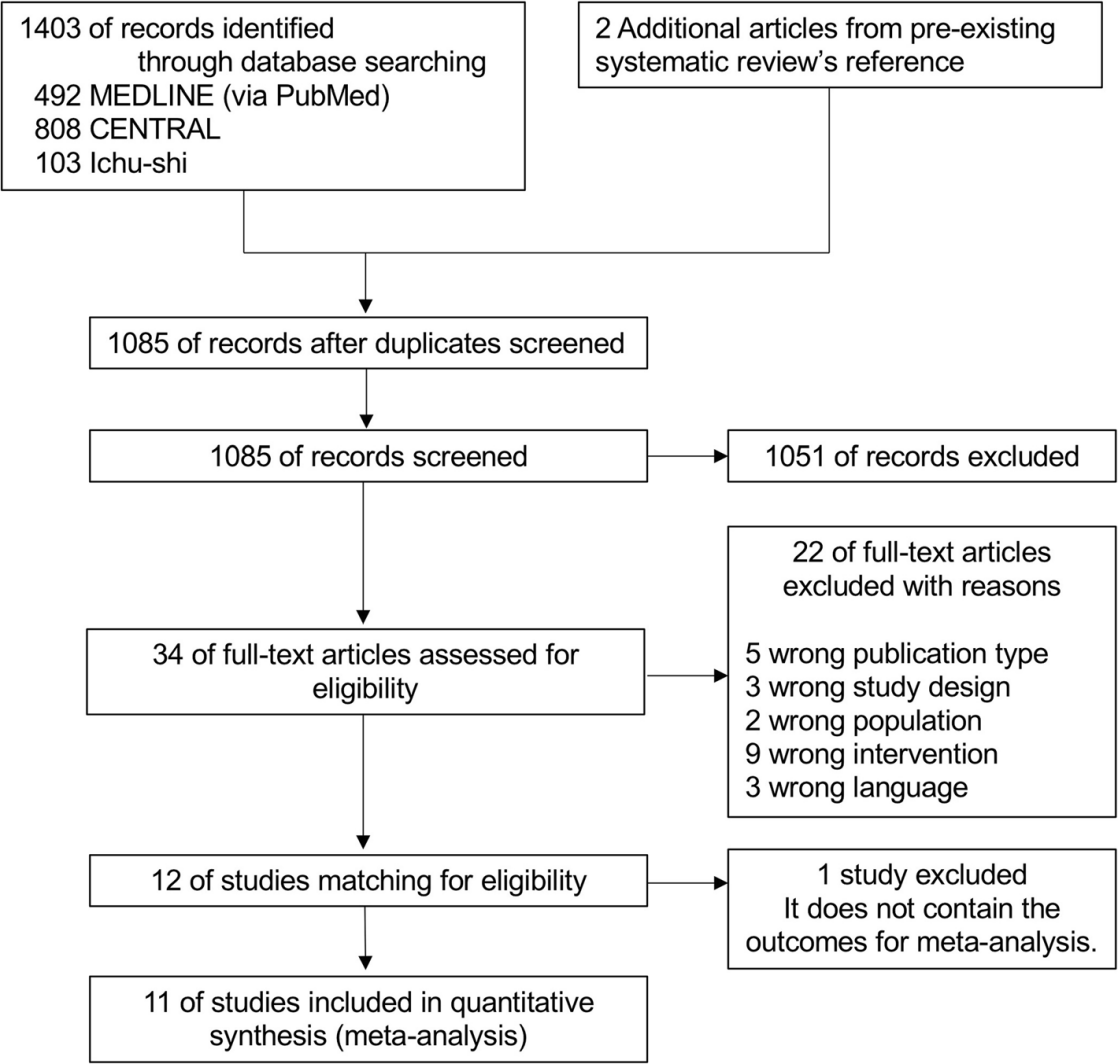
Prisma flowchart

We found 492 studies in MEDLINE via PubMed, 808 in CENTRAL, 103 in Ichu-shi, and 2 in the hand search of the pre-existing systematic review [4]. After excluding duplicate studies, we screened 1085 titles/abstracts. Of these, 34 studies were eligible for a full-text review. After excluding 22 articles, we identified 12 full-text articles that met the eligibility criteria for qualitative synthesis. We excluded one article [13] owing to the lack of information on primary and secondary outcomes for meta-analysis. Finally, we performed a meta-analysis on 11 studies [14–24] (Fig. 1).

### Included studies

The patient characteristics and the description of intervention and control of the included 11 studies are summarized in Tables 1 and 2. Among 1322 included patients, 662 (50.1%) were assigned to early mobilization (intervention). All studies compared early mobilization versus usual care or delayed mobilization.

**Table 1:** Patient characteristics in the included trials

Authors	Year	Target population	Number of patients	Sex (male)	Age	Physiological severity
			I/C	I/C	I/C	I/C
Patman [14]	2001	Adult patients who underwent cardiac surgery in SICU	101/109	81/77	62.8 (12.2)/67.3 (14.4)	-/-
Schweickert [15]	2009	Sedated adult patients with MV in the ICU	49/55	20/22	57.7 (36.3-69.1)/54.4 (46.5-66.4)	20 (15.8-24.0)/19 (13.3-23.0)*
Brummel [16]	2014	Adult patients with respiratory failure and/or shock in ICU	22/22	13/8	62 (48-67)/60 (51-69)	21.5 (20.0-28.8)/27 (17.5-31.0) *
Kayambu [17]	2015	Critically ill adult patients admitted to ICU with sepsis	26/24	18/14	62.5 (30-83)/65.5 (37-85)	28 (7.6)/27 (6.8) *
Morris [18]	2016	Adult patients admitted to the ICU with MV	150/150	66/68	55 (17)/58 (14)	76 (26)/75 (27) †
Moss [19]	2016	Adult patients who required MV	59/61	36/35	56 (14)/49 (15)	17.9 (6.2)/17.4 (5.6) *
Schaller [20]	2016	Adult patients with MV in SICU	104/96	65/61	66 (48-73)/64 (45-76)	16 (12-22)/17 (11-22) *
Dong Z [21]	2016	Adult patients who underwent CABG	53/53	20/22	62.6 (12.8)/60.2 (15.1)	16.3 (4.2)/17.2 (4.3) *
Hodgson [22]	2016	Critically ill adult patients with MV in ICU	29/21	21/9	64 (12)/53 (15)	19.8 (9.8)/15.9 (6.9) *
Maffei [23]	2017	Liver transplant recipients in ICU	20/20	15/16	54 (9)/52 (9)	43 (14)/45 (12) ‡
Moradian [24]	2017	Adult patients who underwent CABG	49/49	33/30	59 (10)/60 (11.3)	-/-

Age and physiological severity are described as mean (SD) or median (IQR). Sex is described as the number of men in each group. *APACHE2 †APACHE3, ‡SAPS2. *I/C* intervention/control, *APACHE2 or 3* Acute Physiology and Chronic Health Evaluation 2 or 3 score, *SAPS2* Simplified Acute Physiology Score, *ICU* intensive care unit, *MV* mechanical ventilation, *SICU* surgical ICU, *CABG* coronary artery bypass graft

**Table 2:** Description of intervention and control groups

Authors	Intervention		Control	
	Contents	Standardized protocol	Contents	Standardized protocol
Patman [14]	Rehabilitation during the intubation period	No	No rehabilitation during the intubated	-
Schweickert [15]	Early exercise and mobilization	Yes	Usual care	No
Brummel [16]	Early once-daily PT	Yes	Usual care	Yes
Kayambu [17]	Early targeted physical rehabilitation program	Yes	Usual care	No
Morris [18]	Standardized rehabilitation therapy	Yes	Usual care	No
Moss [19]	Intensive PT program	Yes	Usual care	No
Schaller [20]	Early, goal-directed mobilization	Yes	Usual care	Yes
Dong Z [21]	Rehabilitation beginning in ICU	No	No mobilization in ICU	-
Hodgson [22]	Early goal-directed mobilization algorithm	Yes	Usual care	No
Maffei [23]	Early and intensive rehabilitation	Yes	Usual care	No
Moradian [24]	Mobilization in POD1	Yes	Usual care	No

*PT* physiotherapy, *ICU* intensive care unit, *POD* post-operative day

### Risk of bias assessment

**Fig. 2:**
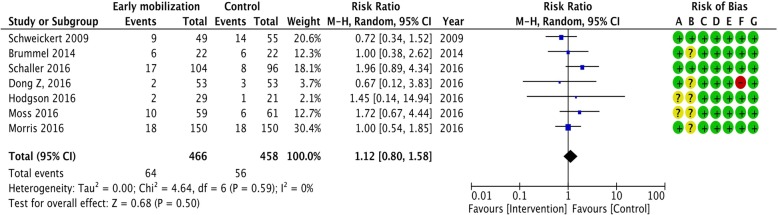
Summary of meta-analysis and risk of bias for in-hospital mortality. CI: confidence interval, M–H: Mantel–Haenszel method. Risk of bias legend: (A) random sequence generation (selection bias), (B) allocation concealment (selection bias), (C) blinding of participants and personnel (performance bias), (D) blinding of outcome assessment (detection bias), (E) incomplete outcome data (attrition bias), (F) selective reporting (reporting bias, (G) other bias

**Fig. 3:**
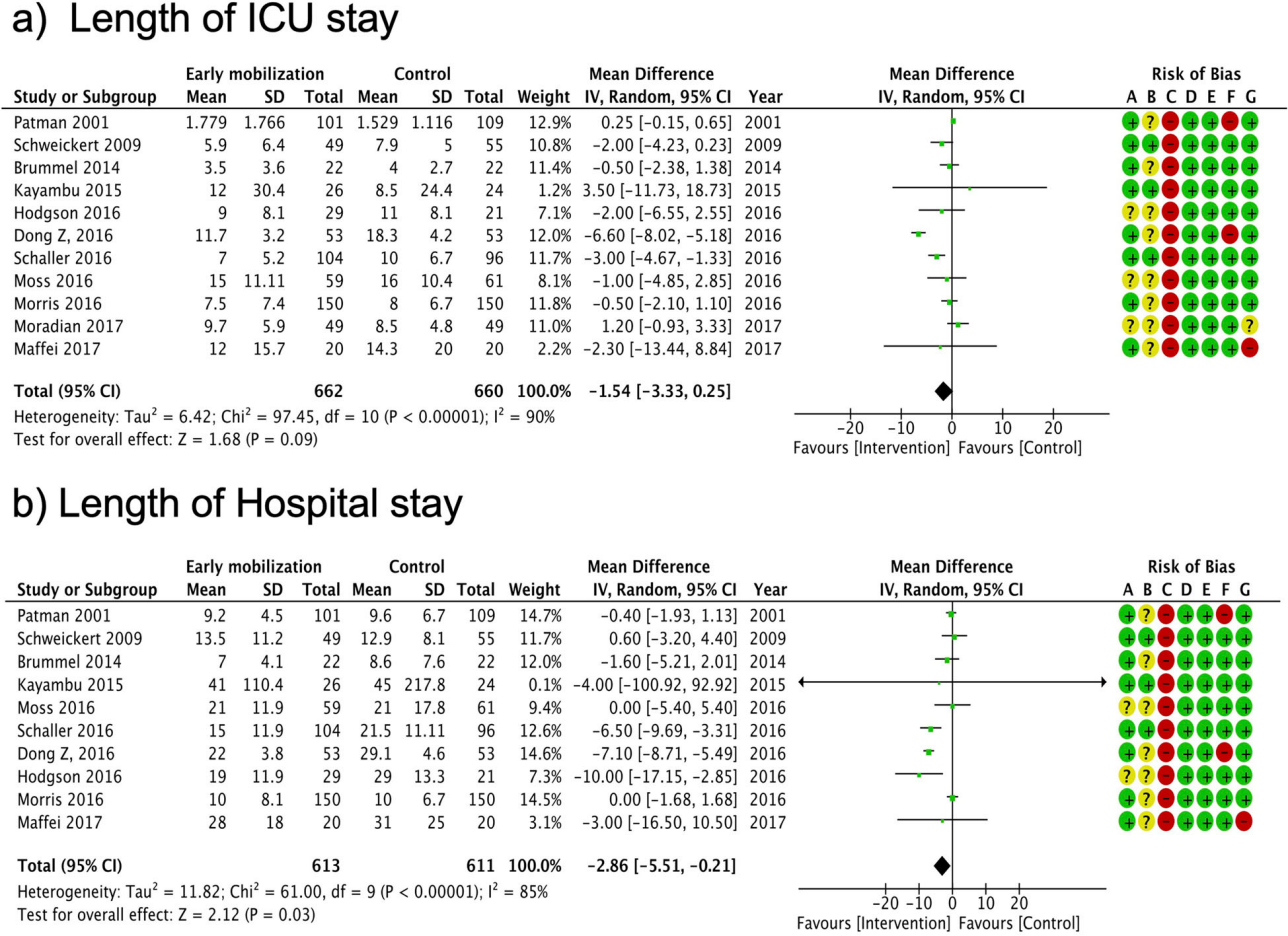
Summary of meta-analysis and risk of bias for length of stay. **a** Length of ICU stay, **b** length of hospital stay. CI confidence interval, M–H Mantel–Haenszel method, ICU intensive care unit. Risk of bias legend: (A) random sequence generation (selection bias), (B) allocation concealment (selection bias), (C) blinding of participants and personnel (performance bias), (D) blinding of outcome assessment (detection bias), (E) incomplete outcome data (attrition bias), (F) selective reporting (reporting bias), (G) other bias

**Fig. 4:**
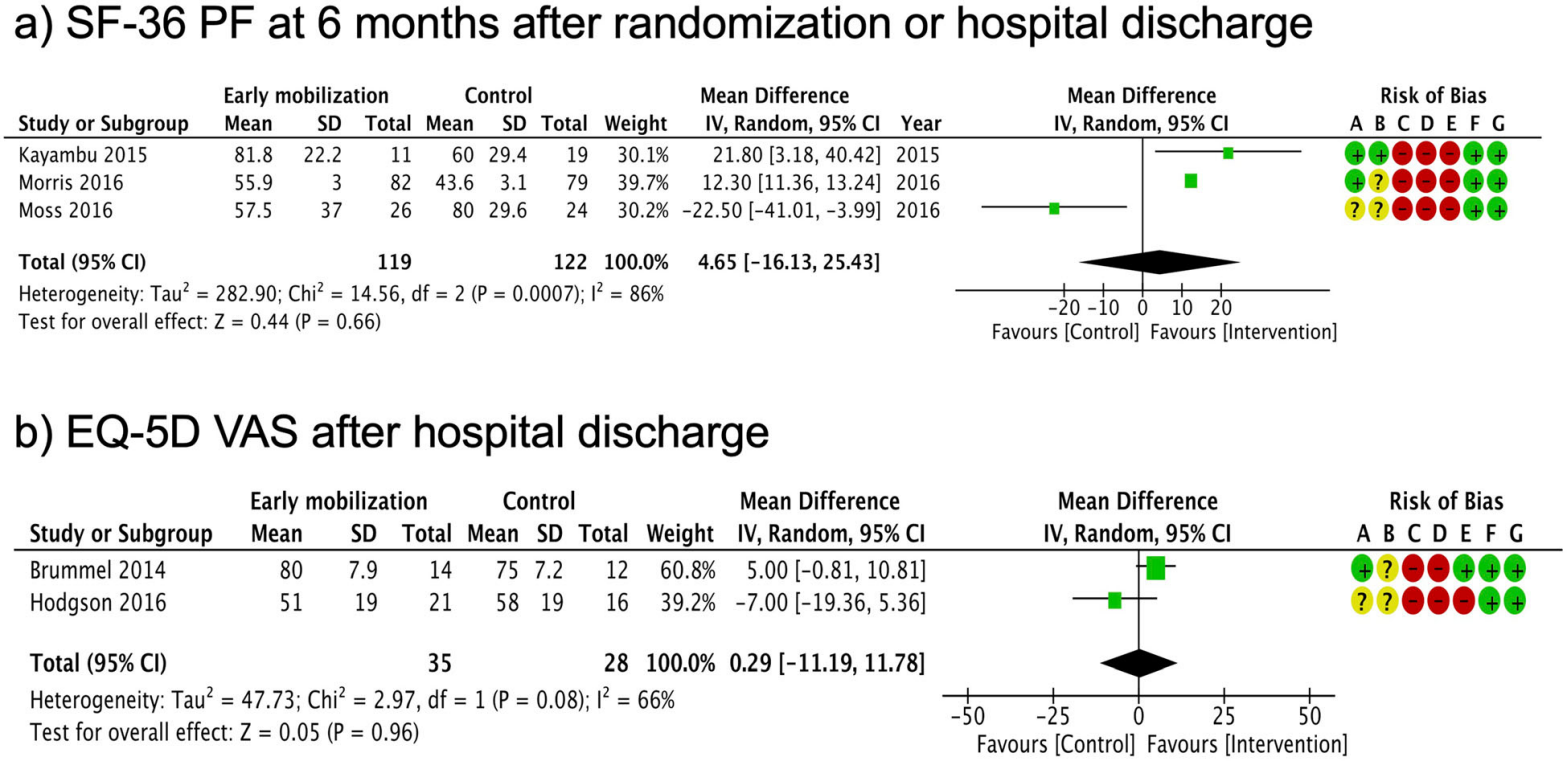
Summary of meta-analysis and risk of bias for health-related QOL (SF-36PF, EQ-5D VAS). **a** SF-36 PF at 6 months after randomization or hospital discharge. **b** EQ-5D VAS after hospital discharge. CI confidence interval, M–H Mantel–Haenszel method, QOL quality of life, EQ-5D EuroQol 5 Dimension, SF-36 PF Short Form Health Survey 36-Item, VAS visual analog scale. Risk of bias legend: (A) random sequence generation (selection bias), (B) allocation concealment (selection bias), (C) blinding of participants and personnel (performance bias), (D) blinding of outcome assessment (detection bias), (E) incomplete outcome data (attrition bias), (F) selective reporting (reporting bias), (G) other bias

**Fig. 5:**
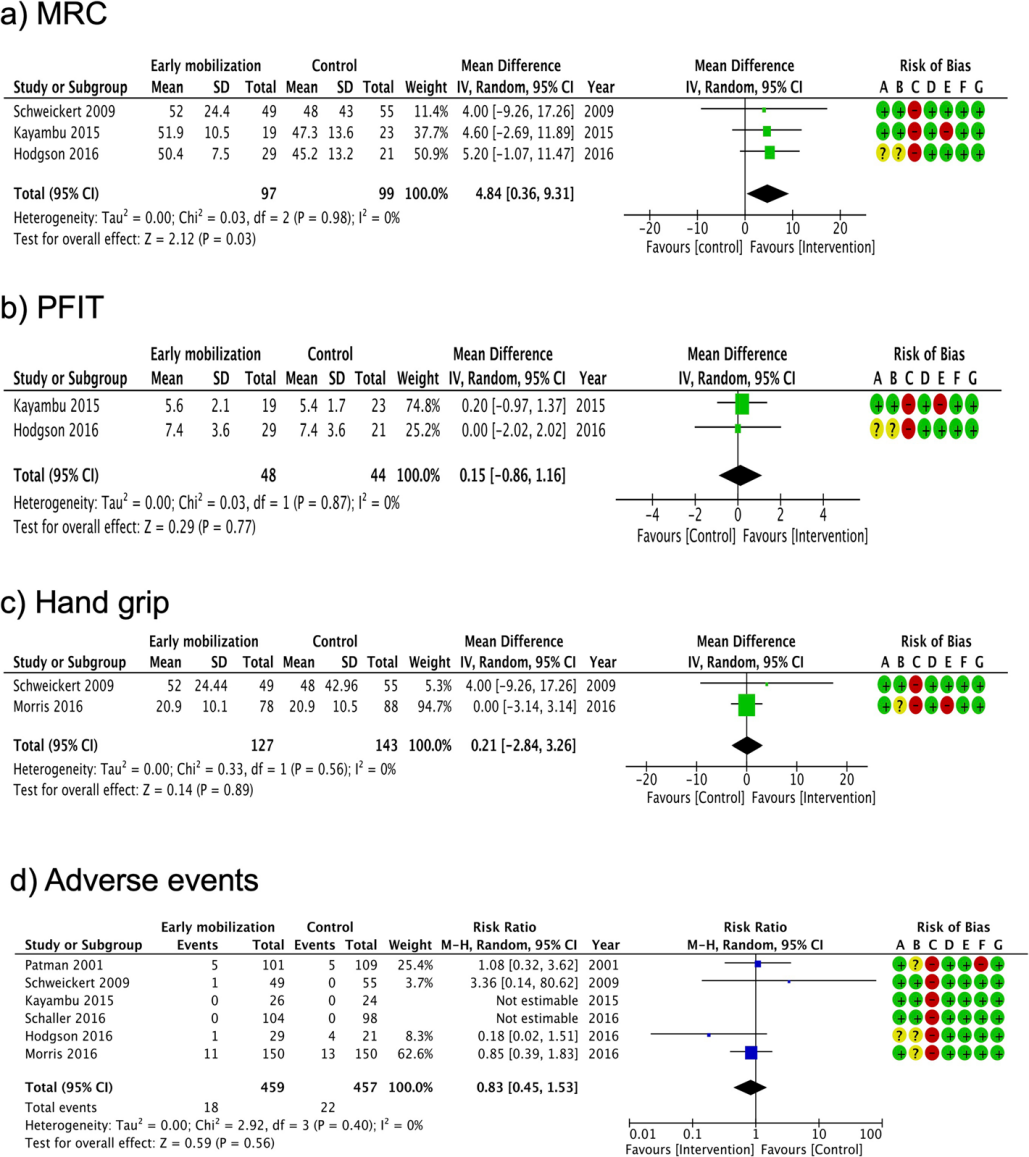
Summary of meta-analysis and risk of bias for secondary outcomes: **a** MRC, **b** PFIT, **c** hand grip, and **d** adverse events. MRC Medical Research Council Scale for Muscle Strength, PFIT Physical Function in ICU Test, CI confidence interval, M–H Mantel–Haenszel method. Risk of bias legend: (A) random sequence generation (selection bias), (B) allocation concealment (selection bias), (C) blinding of participants and personnel (performance bias), (D) blinding of outcome assessment (detection bias), (E) incomplete outcome data (attrition bias), (F) selective reporting (reporting bias), (G) other bias

Figures 2, 3, 4, and 5 summarize the risk of bias assessments [green (+): low risk, red (-): high risk, and yellow (?): unclear]. The details of risk assessments are described in the Additional file 1. We addressed the following domains in our evaluation of bias for this trial: random sequence generation, allocation concealment, selective reporting, and other bias. We assessed the risk of bias in each outcome, particularly regarding the blinding of participants and personnel (performance bias), blinding of outcome assessment (detection bias), and incomplete outcome (attrition bias).

Some studies did not present sufficient information for the assessment of random sequence generation and allocation concealment; therefore, these studies were consequently categorized as “unclear ”[14, 16, 18, 19, 21–24]. With regard to blinding participants and personnel (performance bias), we evaluated the primary outcome of mortality as “low” risk because it was not likely to be influenced by a lack of blinding. For all other primary outcomes, we assessed all studies as “high” risk because it was largely impossible to perform physiotherapy blinded to patients and clinicians. With respect to blinding outcome assessment (detection bias), we scored subjective outcomes such as health-related QOL as “high” risk because the outcome assessor was not blinded to the allocation. For other objective outcomes such as physical function, if the outcome assessor was appropriately blinded, we scored the outcome as “low” risk. For incomplete outcome, we assessed the outcome as “low” risk if the proportion of missing outcome data was small enough or if the reasons for missing outcome data were unlikely to be related to true outcome. Otherwise, we evaluated the risk as “high.” Furthermore, for selective reporting, we scored two studies [14, 21] as “high” risk because we could not find their prespecified study protocols. For other biases, one study was evaluated as “high” risk, and another was assessed as “unclear.” The former study was an unblinded trial using blocked randomization and was held at a single institution, and the latter study did not indicate a funding source. (The detail of the risk of bias was described in Additional file 2).

### Meta-analysis of the results

We synthesized the primary outcomes as follows: in-hospital mortality, duration of ICU and in-hospital stay, SF-36 physical function (PF) 6 months after randomization or hospital discharge, and EQ-5D visual analog scale (VAS) after discharge. In the primary outcomes, the pooled RR of early mobilization versus control for in-hospital mortality was 1.12 (95% CI 0.80-1.58, *I*^2^ = 0%) (Fig. 2). The pooled MD for duration of ICU stay was -1.54 (95% CI -3.33 to 0.25, *I*^2^ = 90%) (Fig. 3). The pooled MD for duration of hospital stay was -2.86 (95% CI -5.51 to -0.21, *I*^2^ = 85%) (Fig. 3). The pooled MD for SF-36 PF 6 months after discharge was 4.65 (95% CI -16.13 to 25.43, *I*^2^ = 86%) (Fig. 4). The pooled MD for EQ-5D VAS after discharge was 0.29 (95% CI -11.19 to 11.78, *I*^2^ = 66%) (Fig. 4). According to these data, the effect of early mobilization on primary outcomes was only favorable for the length of hospital stay.

The results of the meta-analysis for secondary outcomes are summarized in Fig. 5. As previous studies have reported, the MRC score in the early mobilization group was superior to that in the control group [pooled MD 4.84 (95% CI: 0.36-9.31)]. In other secondary outcomes, there was no significant difference, suggesting the superiority of early mobilization over control (Fig. 5). We could not perform a meta-analysis for cognitive function (MMSE) and mental disorder because the number of trials available to report these outcomes was less than two.

For assessment of publication bias, we described a funnel plot among the outcomes, which were reported in more than ten trials (the length of ICU and hospital stay). This plot indicated a symmetric distribution of the effect (Additional file 2), and there was no publication bias.

## Discussion

### Summary of the main results

Our systematic review and meta-analysis indicated that there were no apparent differences between early mobilization and control in terms of in-hospital mortality and health-related QOL (SF-36PF and EQ-5D VAS). Early mobilization was favorable in terms of the length of hospital stay; however, there may have been bias due to the lack of blinding or clinical heterogeneity. These results demonstrate that the currently available data are inadequate in establishing the superiority of early mobilization in improving relevant patient outcomes. A larger study is needed to evaluate the effects of early mobilization on in-hospital mortality and health-related QOL in critically ill patients.

### Strengths in relation to other reviews

Compared to previous studies, our systematic review and meta-analysis has two strengths. First, our review updated the best research evidence for the efficacy of early mobilization in ICU patients. Systematic reviews and meta-analyses [4, 5] have already been published in this regard. However, additional RCTs were performed [23, 24] after those studies; it is therefore necessary to update the current literature. These recent trials were included in the 11 trials evaluated in this study; it therefore provides the best updated evidence.

Second, our review focused on patient-relevant outcomes such as mortality and health-related QOL. The previous review [4] did not include mortality as an outcome during meta-analysis. Another recently published review [3] did not perform meta-analysis for health-related QOL outcomes. Conversely, our review provides results for both, mortality and health-related QOL; these results are therefore more clinically relevant than those from previous reviews.

### Interpretation and implications for practice and further research

No apparent differences in in-hospital mortality were noted between the intervention and control groups in our analysis. This may be attributed to the lack of statistical power in detecting the difference, as mortality was relatively low among the eligible patients in the included trials [early mobilization group: 13.7 (64/466) vs. control group: 12.2% (56/458)]. Future research will therefore require considerably larger cohorts to investigate any difference. Our analysis showed early mobilization to be a favorable factor for the length of in-hospital stay and MRC. These findings may provide sufficient evidence to recommend early mobilization in clinical practice. However, the results should be interpreted with caution, as they may be influenced by performance bias owing to a lack of blinding among clinicians. The statistical heterogeneity of in-hospital length of stay in this cohort was substantially high (*I*^2^ = 85%). Despite the lack of apparent baseline imbalances in physiological severity, our study population was clinically heterogeneous (i.e., variable settings and medical conditions); this may have influenced the outcome. Heterogeneity was also high for the health-related QOL outcomes (SF-36 PF: *I*^2^ = 86%, EQ-5D VAS: *I*^2^ = 55%); these may be influenced by a high risk of bias related to performance, detection, and attrition. The risk of performance and detection bias is invariably high in trials of this design; however, the influence of incomplete outcomes can and should be reduced in future research.

### Limitations

This study had several limitations. First, we did not include studies written in languages other than English or Japanese. Thus, trials that were otherwise eligible for inclusion may have been overlooked. Second, some of the trials were pilot or feasibility studies; therefore, the individual sample sizes were limited. Further, the number of included trials was limited in terms of the health-related QOL outcomes. This may have led to a lack of power in detecting any differences in effect. Third, the definition of the intervention was clinically heterogeneous. These limitations should be considered while interpreting the study results.

## Conclusions

This systematic review and meta-analysis demonstrated no apparent differences between early mobilization and usual care regarding in-hospital mortality and health-related QOL (SF-36PF and EQ-5D VAS) among critically ill patients in the ICU. This suggests that currently available data are inadequate for evaluating the effect of early mobilization on relevant patient outcomes. Larger studies are warranted in the future for detailed evaluation of the effects of early mobilization on in-hospital mortality and health-related QOL in critically ill patients.

## Supplementary information

**Additional file 1.** Study protocol

**Additional file 2.** Detail of the risk of bias

## Data Availability

Not applicable

## Abbreviations

CI: confidence interval
EQ-5D: EuroQol 5 Dimension
ICU: intensive care unit
ICU-AW: ICU-acquired weakness
MD: mean difference
M–H: Mantel–Haenszel method
MRC: Medical Research Council Scale for Muscle Strength score
PFIT: the Physical Function in ICU Test
QOL: quality of life
RCT: randomized clinical trial
RR: risk ratio
SF-36 PF: Short Form Health Survey 36-Item
SF-36: the Short Form Health Survey 36-Item
VAS: visual analog scale
