# Supplementary material for: Early versus delayed mobilization for in-hospital mortality and health-related quality of life among critically ill patients: a systematic review and meta-analysis
Source: J Intensive Care. 2019 Dec 9;7:57. doi: 10.1186/s40560-019-0413-1 (PMC6902574; doi:10.1186/s40560-019-0413-1)
Supplement: Supplementary file 1 — Additional file 1. Study protocol [file 40560_2019_413_MOESM1_ESM.docx]

**Additional file 1**

**Study protocol**

**Title: The effect of early mobilization among the critically ill patients; Systematic review of randomized controlled trial**

P: Critically ill patients admitted to critical care unit

I: Early mobilization started within one week of ICU admission

C: Usual care (or later mobilization than the intervention group)

O1: Mortality

O2: The length of ICU stay /hospital stay

O3: Quality of Life (SF-36, EQ5D)

**Anticipated or actual start date.**

April 12, 2019

**Anticipated completion date**

July 21, 2019

**Review team members and their organisational affiliations.**

Takeshi Unoki: Sapporo City University

Yohei Okada: Kyoto University, Graduate school of medicine

Yujiro Matsuishi: University of Tsukuba

Yuko Egawa: Saitama Red Cross Hospital

**Funding source**

None

**Conflict of interest**

None

**Collaborators**

Kei Hayashida: The Feinstein Institute for Medical Research

Shigeaki Inoue: Kobe University, Graduate School of Medicine

**Review question**

Our research question is whether earlier mobilization should be performed to prevent the post intensive care syndrome.

**Searches**

We searched for eligible trials in the following databases: Cochrane Central Register of Controlled Trials (CENTRAL) in the Cochrane Library, MEDLINE via Pubmed, and Igaku-Chuo-Zasshi.

We limited studies based on English or Japanese language of publication. We applied the Cochrane highly sensitive filter for randomized controlled trials in MEDLINE.

**Condition or domain being studied**

Early mobilization in critical care unit

**Participants/population**

Adult (aged ≥18 years) critically ill patients admitted to critical care unit

**Interventions**

Early mobilization defined as physical therapy or occupational therapy which was started earlier than the usual care or control within one week of ICU admission.

**Comparator**

Usual care or mobilization program started later than the intervention

**Type of study to be included**

Randomized controlled trial will be included.

**Main outcome**

O1: Mortality

O2: The length of ICU stay /hospital stay

O3: Quality of Life (SF-36, EQ5D)

**Additional outcome**

Q4: Physical function such as grip strength, MRC score or PEIT

Q5: Cognitive function after discharge

Q6: Mental disorder such as depression or anxiety

Q7: All adverse events

**Data extraction**

We will design forms for trial inclusion or exclusion, data extraction and for requesting additional published information from authors of the original reports. The review authors will perform data extraction independently using specifically designed paper forms that are piloted and improved for data extraction from identified eligible trials. We will compare the extracted data for differences, which we resolved by discussion.

**Risk of assessment**

We will use the Cochrane risk of bias tool to assess the quality of study design and the extent of potential bias by considering the domains of this bias tool.

**Analysis of sub-group**

**Strategy for data synthesis**

We will perform meta-analysis according to the "Cochrane Handbook for Systematic Reviews of Interventions" and PRISMA (Preferred Reporting Items for Systematic Reviews and Meta-Analyses) guidelines by using software Review Manager (RevMan 5.3). Estimates will be pooled using a random effects model. To assess for between-study heterogeneity, the Cochran Q statistic will be calculated and I-squared used to quantify the magnitude of between-study heterogeneity. If significant heterogeneity is found, the median of the estimates will be reported rather than a weighted, pooled estimate.

**Type and method of review**

-Intervention

-Meta-analysis
