# Supplementary material for: Early versus delayed mobilization for in-hospital mortality and health-related quality of life among critically ill patients: a systematic review and meta-analysis
Source: J Intensive Care. 2019 Dec 9;7:57. doi: 10.1186/s40560-019-0413-1 (PMC6902574; doi:10.1186/s40560-019-0413-1)
Supplement: Supplementary file 2 — Additional file 2. Detail of the risk of bias [file 40560_2019_413_MOESM2_ESM.docx]

**Additional file 2**

**Detail of the risk of bias**

**For the all outcomes**

| Author | Year | Risk of Bias | | | |
| --- | --- | --- | --- | --- | --- |
|  |  | Random sequence generation | Allocation concealment | Selective reporting | Other bias |
| Patman[1] | 2001 | Low | Unclear*1 | High*2 | Low |
| Schweickert[2] | 2009 | Low | Low | Low | Low |
| Brummel[3] | 2014 | Low | Unclear*3 | Low | Low |
| Kayambu[4] | 2015 | Low | Low | Low | Low |
| Morris[5] | 2016 | Low | Unclear*4 | Low | Low |
| Moss[6] | 2016 | Unclear*5 | Unclear*6 | Low | Low |
| Schaller[7] | 2016 | Low | Low | Low | Low |
| Dong Z[8] | 2016 | Low | Unclear*7 | High*8 | Low |
| Hodgson[9] | 2016 | Unclear*9 | Unclear*10 | Low | Low |
| Maffei[10] | 2017 | Low | Unclear*11 | Low | High*12 |
| Moradian[11] | 2017 | Unclear*13 | Unclear*14 | Low | Unclear*15 |

**Reasons**

*1 Unclear description about the allocation concealment

*2 Unavailable prespecified protocol

*3 Unclear description about the allocation concealment

*4 Unclear description about the allocation concealment

*5 Unclear description about the random sequence generation

*6 Unclear description about the allocation concealment

*7 Unclear description about the allocation concealment

*8 Unavailable prespecified protocol

*9 Unclear description about the random sequence generation

*10 Unclear description about the allocation concealment

*11 Unclear description about the allocation concealment

*12 Blocked randomization performed in unblinded trial at the single institution

*13 Unclear description about the random sequence generation

*14 Unclear description about the allocation concealment

*15 Unclear statement about funding source

**For in-hospital mortality**

| Author | Year | Risk of Bias | | |
| --- | --- | --- | --- | --- |
|  |  | Blinding of participants and personnel*1 | Blinding of outcome assessment*2 | Incomplete outcome |
| Schweickert[2] | 2009 | Low | Low | Low |
| Brummel[3] | 2014 | Low | Low | Low |
| Morris[5] | 2016 | Low | Low | Low |
| Moss[6] | 2016 | Low | Low | Low |
| Schaller[7] | 2016 | Low | Low | Low |
| Dong Z[8] | 2016 | Low | Low | Low |
| Hodgson[9] | 2016 | Low | Low | Low |

*1,2 Mortality is not likely to be influenced by lack of blinding.

**For length of ICU and hospital stay**

| Author | Year | Risk of Bias | | |
| --- | --- | --- | --- | --- |
|  |  | Blinding of participants and personnel*1 | Blinding of outcome assessment | Incomplete outcome |
| Patman[1] | 2001 | High | Low | Low |
| Schweickert[2] | 2009 | High | Low | Low |
| Brummel[3] | 2014 | High | Low | Low |
| Kayambu[4] | 2015 | High | Low | Low |
| Morris[5] | 2016 | High | Low | Low |
| Moss[6] | 2016 | High | Low | Low |
| Schaller[7] | 2016 | High | Low | Low |
| Dong Z[8] | 2016 | High | Low | Low |
| Hodgson[9] | 2016 | High | Low | Low |
| Maffei[10] | 2017 | High | Low | Low |
| Moradian[11] | 2017 | High | Low | Low |

*1 It is generally impossible to perform the physiotherapy blinded to the patients and clinicians.

**For health-related QOL (SF-36)**

| Author | Year | Risk of Bias | | |
| --- | --- | --- | --- | --- |
|  |  | Blinding of participants and personnel*1 | Blinding of outcome assessment*2 | Incomplete outcome |
| Kayambu[4] | 2015 | High | High | High*3 |
| Morris[5] | 2016 | High | High | High*4 |
| Moss[6] | 2016 | High | High | High*5 |

*1 It is generally impossible to perform the physiotherapy blinded to the patients and clinicians.

*2 It is generally impossible to perform the physiotherapy blinded to outcome assessors (patients).

*3 Many patients were lost for follow-up without obvious reasons.

*4 Many patients were lost for follow-up with unclear reasons.

*5 Many patients were lost for follow-up with unclear reasons.

**For health-related QOL (EQ-5D)**

| Author | Year | Risk of Bias | | |
| --- | --- | --- | --- | --- |
|  |  | Blinding of participants and personnel*1 | Blinding of outcome assessment*2 | Incomplete outcome |
| Brummel[3] | 2014 | High | High | Low |
| Hodgson[9] | 2016 | High | High | High*3 |

*1 It is generally impossible to perform the physiotherapy blinded to the patients and clinicians.

*2 It is generally impossible to perform the physiotherapy blinded to outcome assessors (patients).

*3 No description about the reasons for lost follow-up

**For PFIT**

| Author | Year | Risk of Bias | | |
| --- | --- | --- | --- | --- |
|  |  | Blinding of participants and personnel*1 | Blinding of outcome assessment | Incomplete outcome |
| Kayambu[4] | 2015 | High | Low | High*2 |
| Hodgson[9] | 2016 | High | Low | Low |

*1 It is generally impossible to perform the physiotherapy blinded to the patients and clinicians.

*2 No description about the reason for lost follow-up

**For MRC**

| Author | Year | Risk of Bias | | |
| --- | --- | --- | --- | --- |
|  |  | Blinding of participants and personnel*1 | Blinding of outcome assessment | Incomplete outcome |
| Schweickert[2] | 2009 | High | Low | Low |
| Kayambu[4] | 2015 | High | Low | High*2 |
| Hodgson[9] | 2016 | High | Low | Low |

*1 It is generally impossible to perform the physiotherapy blinded to the patients and clinicians.

*2 No description about the reason for lost follow-up

**For hand grip strength**

| Author | Year | Risk of Bias | | |
| --- | --- | --- | --- | --- |
|  |  | Blinding of participants and personnel*1 | Blinding of outcome assessment | Incomplete outcome |
| Schweickert[2] | 2009 | High | Low | Low |
| Morris[5] | 2016 | High | Low | High*2 |

*1 It is generally impossible to perform the physiotherapy blinded to the patients and clinicians.

*2 Missing data might have influenced the imbalance between intervention and control.

**For adverse outcome**

| Author | Year | Risk of Bias | | |
| --- | --- | --- | --- | --- |
|  |  | Blinding of participants and personnel*1 | Blinding of outcome assessment*2 | Incomplete outcome |
| Patman[1] | 2001 | High | Low | Low |
| Schweickert[2] | 2009 | High | Low | Low |
| Kayambu[4] | 2015 | High | Low | Low |
| Morris[5] | 2016 | High | Low | Low |
| Schaller[7] | 2016 | High | Low | Low |
| Hodgson[9] | 2016 | High | Low | Low |

*1 It is generally impossible to perform the physiotherapy blinded to the patients and clinicians.

*2 It is generally impossible to perform the physiotherapy blinded to the patients and clinicians, however, the detection for the prespecified objective adverse events is unlikely to be influenced by the blinding.

**Funnel plot**

For length of ICU stay

For length of hospital stay

SE: standard error, MD: mean difference

Supplementary Reference

1. Patman S, Sanderson D, Blackmore M: **Physiotherapy following cardiac surgery: is it necessary during the intubation period?** In: *Aust J Physiother. Volume 47*, edn. Australia; 2001: 7-16.

2. Pohlman MC, Schweickert WD, Pohlman AS, Nigos C, Pawlik AJ, Esbrook CL, Spears L, Miller M, Franczyk M, Deprizio D *et al*: **Feasibility of physical and occupational therapy beginning from initiation of mechanical ventilation**. *Crit Care Med* 2010, **38**(11):2089-2094.

3. Brummel NE, Girard TD, Ely EW, Pandharipande PP, Morandi A, Hughes CG, Graves AJ, Shintani A, Murphy E, Work B *et al*: **Feasibility and safety of early combined cognitive and physical therapy for critically ill medical and surgical patients: the Activity and Cognitive Therapy in ICU (ACT-ICU) trial**. *Intensive Care Med* 2014, **40**(3):370-379.

4. Kayambu G, Boots R, Paratz J: **Early physical rehabilitation in intensive care patients with sepsis syndromes: a pilot randomised controlled trial**. *Intensive Care Med* 2015, **41**(5):865-874.

5. Morris PE, Berry MJ, Files DC, Thompson JC, Hauser J, Flores L, Dhar S, Chmelo E, Lovato J, Case LD *et al*: **Standardized Rehabilitation and Hospital Length of Stay Among Patients With Acute Respiratory Failure: A Randomized Clinical Trial**. *JAMA* 2016, **315**(24):2694-2702.

6. Moss M, Nordon-Craft A, Malone D, Van Pelt D, Frankel SK, Warner ML, Kriekels W, McNulty M, Fairclough DL, Schenkman M: **A Randomized Trial of an Intensive Physical Therapy Program for Patients with Acute Respiratory Failure**. *Am J Respir Crit Care Med* 2016, **193**(10):1101-1110.

7. Schaller SJ, Anstey M, Blobner M, Edrich T, Grabitz SD, Gradwohl-Matis I, Heim M, Houle T, Kurth T, Latronico N *et al*: **Early, goal-directed mobilisation in the surgical intensive care unit: a randomised controlled trial**. *Lancet* 2016, **388**(10052):1377-1388.

8. Dong Z, Yu B, Zhang Q, Pei H, Xing J, Fang W, Sun Y, Song Z: **Early Rehabilitation Therapy Is Beneficial for Patients With Prolonged Mechanical Ventilation After Coronary Artery Bypass Surgery**. *Int Heart J* 2016, **57**(2):241-246.

9. Hodgson CL, Bailey M, Bellomo R, Berney S, Buhr H, Denehy L, Gabbe B, Harrold M, Higgins A, Iwashyna TJ *et al*: **A Binational Multicenter Pilot Feasibility Randomized Controlled Trial of Early Goal-Directed Mobilization in the ICU**. *Crit Care Med* 2016, **44**(6):1145-1152.

10. Maffei P, Wiramus S, Bensoussan L, Bienvenu L, Haddad E, Morange S, Fathallah M, Hardwigsen J, Viton JM, Le Treut YP *et al*: **Intensive Early Rehabilitation in the Intensive Care Unit for Liver Transplant Recipients: A Randomized Controlled Trial**. *Arch Phys Med Rehabil* 2017, **98**(8):1518-1525.

11. Moradian ST, Najafloo M, Mahmoudi H, Ghiasi MS: **Early mobilization reduces the atelectasis and pleural effusion in patients undergoing coronary artery bypass graft surgery: A randomized clinical trial**. *J Vasc Nurs* 2017, **35**(3):141-145.
